# Supplementary figures and images for: Robust and memory-less median estimation for real-time spike detection
Source: PLoS One. 2024 Nov 26;19(11):e0308125. doi: 10.1371/journal.pone.0308125 (PMC11594402; doi:10.1371/journal.pone.0308125)

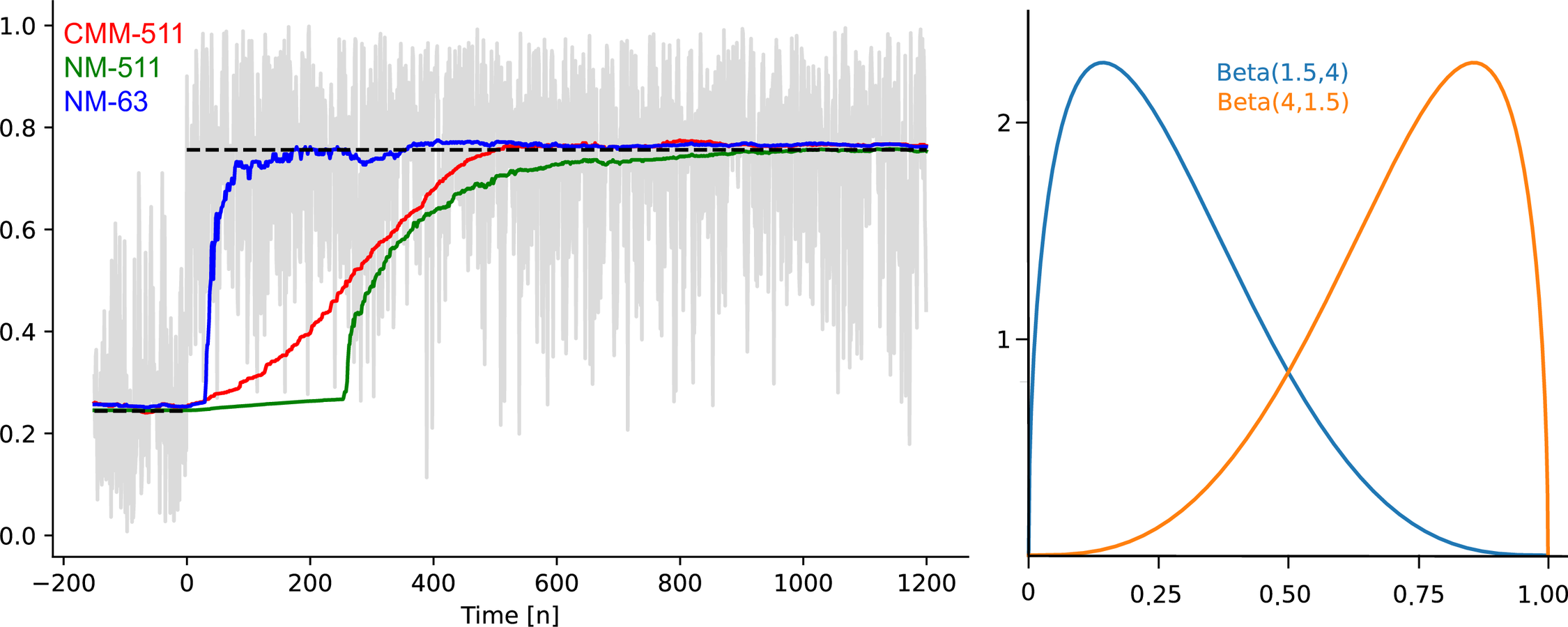

Supplement: S1 Fig — Step median change, from a Beta(1.5, 4) distribution, which has a median of 0.2439 and a mean of 0.2727 (skewed), to a Beta(4, 1.5) distribution, with a median of 0.7561 and a mean of 0.7273. In response to this significant median change, the new median experiences an intrinsic delay of L/2 before it begins to displace older values from the central position of the buffer. In contrast, the CMM exhibits a gradual change characteristic of a sliding window median. Nevertheless, both NM-511 and CMM-511 converge towards the new median value in approximately L time steps (see supplementary S3 Fig). However, NM-63 achieves the same variance estimator as CMM-511, with a significantly faster settling time. (TIF) [file pone.0308125.s001.tif]

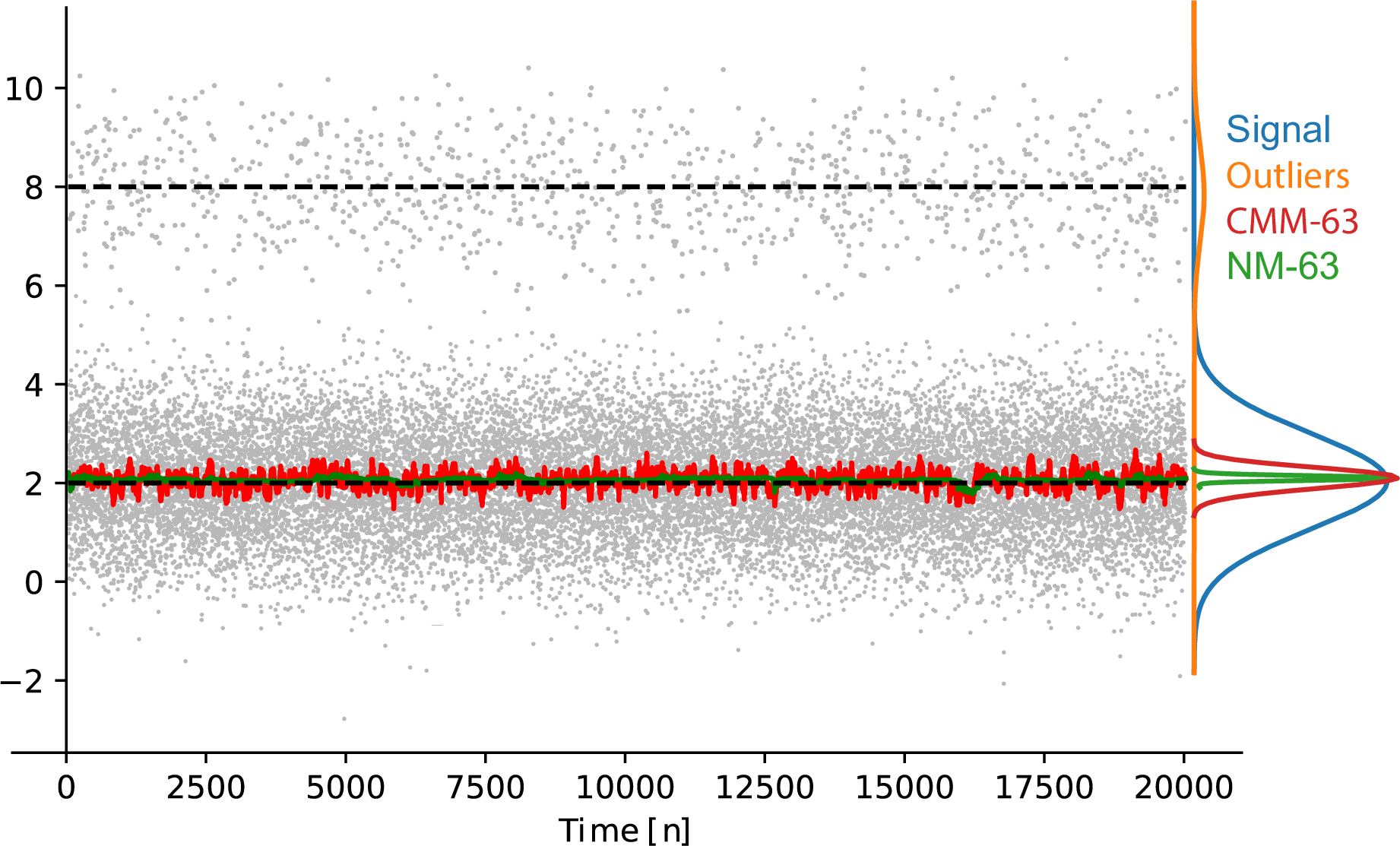

Supplement: S2 Fig — Samples were drawn from a Gaussian mixture that includes both the primary distribution and an outlier distribution (noise). Both estimators demonstrate robustness against extreme outliers. The NM-63 outperforms the CMM-63, exhibiting an estimator variance that is eight times smaller. (TIF) [file pone.0308125.s002.tif]

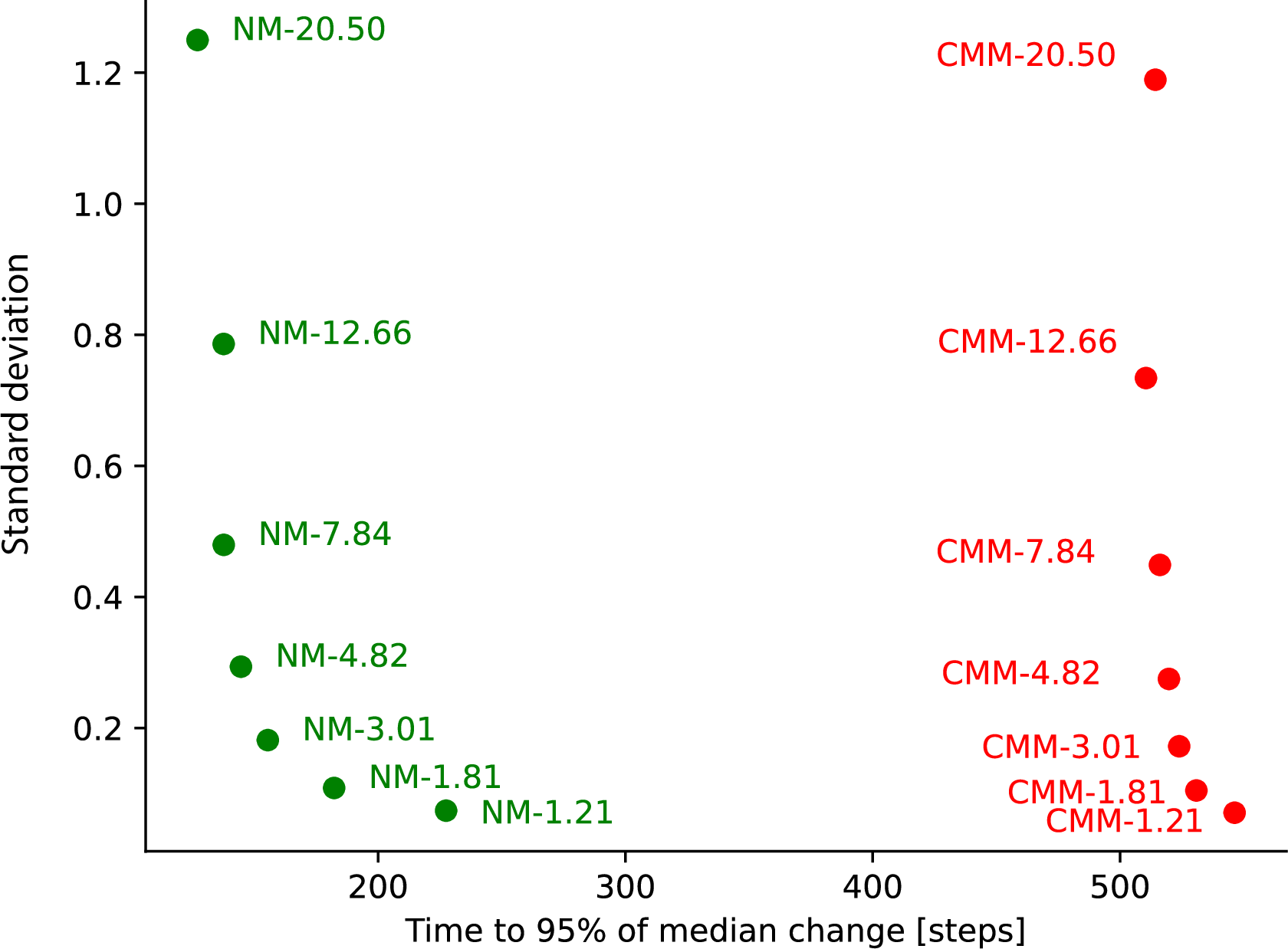

Supplement: S3 Fig — A step change was generated in the median, increasing from 0.67 to 1.21, 1.81, 3.01, 4.82, 7.84, 12.66, and 20.50. These medians correspond to the standard deviation of Gaussian noise, as described by Eq (2). We selected NM-63 and CMM-511 because they exhibit similar estimator variance. While CMM demonstrates a consistent settling time across all levels of step change, which is closer to its buffer length of 511, the settling time of NM is more influenced by the magnitude of the step change. Nevertheless, NM outperforms CMM at all levels, and with moderate step changes, it begins to approach the settling time of L steps. For each configuration, there were 3,000 iterations. The settling time and standard deviation were defined as explained for Fig 4c. (TIF) [file pone.0308125.s003.tif]

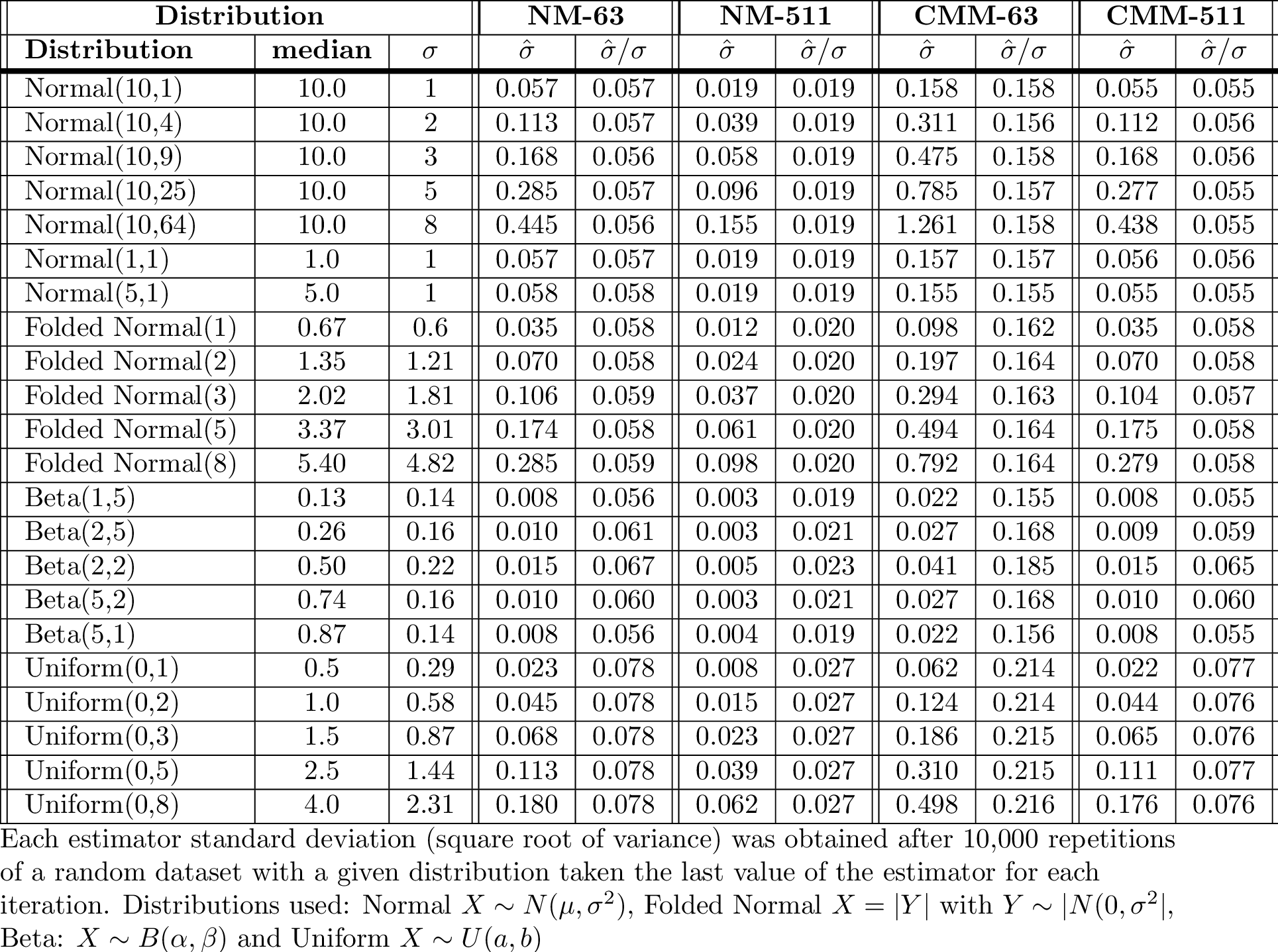

Supplement: S1 Table — The standard deviation of the estimator for various input distributions was analyzed. Each type of distribution was tested with different parameters, revealing that the relationship between the estimator’s standard deviation, denoted as σ^, and the distribution’s standard deviation, σ, remains consistent across all distribution types and is solely dependent on the buffer length. We tested NM and CMM with buffer lengths of 63 and 511 to demonstrate that NM-63 performs equivalently to CMM-511 across all distribution types. (TIF) [file pone.0308125.s004.tif]
