# Supplementary material for: Robust and memory-less median estimation for real-time spike detection
Source: PLoS One. 2024 Nov 26;19(11):e0308125. doi: 10.1371/journal.pone.0308125 (PMC11594402; doi:10.1371/journal.pone.0308125)
Supplement: Attachment — Submitted filename: Plus_One.docx [file pone.0308125.s005.docx]

1. Need more analysis
2. Literature survey is very less in the paper
3. At least 20 latest references need to add in the paper
4. Highlights should be there for main work
5. Comparison with traditional algorithms is impressive so need to design with proper table and diagram.
